# Canine Leishmaniasis in Europe over the Last Decade: A Review of Geographic Trends and Epidemiological Data

**DOI:** 10.3390/pathogens14111082

**Published:** 2025-10-23

**Authors:** Anamaria Plesko, Tiana Florea, Mirela Imre, Diana Hoffman, Ioan Cristian Dreghiciu, Alexandra Pocinoc, Florica Morariu, Ana-Maria Marin, Narcisa Mederle, Gheorghe Darabus, Ion Oprescu, Marius Stelian Ilie, Sorin Morariu

**Affiliations:** 1 Department of Parasitology and Parasitic Disease, Faculty of Veterinary Medicine, University of Life Sciences “King Mihai I” from Timisoara, 119, Calea Aradului, 300645 Timisoara, Romania; mirela.imre@usvt.ro (M.I.); diana.hoffman@usvt.ro (D.H.); cristian.dreghiciu@usvt.ro (I.C.D.); alexandra.pocinoc@usvt.ro (A.P.); anamaria.marin@usvt.ro (A.-M.M.); narcisamederle@usvt.ro (N.M.); gheorghedarabus@usvt.ro (G.D.); ionoprescu@usvt.ro (I.O.); mariusilie@usvt.ro (M.S.I.); sorinmorariu@usvt.ro (S.M.); 2 Department of Biotechnology, Faculty of Bioengineering of Animal Resources, University of Life Sciences “King Mihai I” from Timisoara, 300645 Timisoara, Romania; floricamorariu@usvt.ro

**Keywords:** canine leishmaniasis, Europe, prevalence, vector-borne diseases

## Abstract

Canine leishmaniasis is an emerging threat in Europe shaped by ecological, climatic, and socio-economic factors. This systematic review examines the prevalence of *Leishmania* spp. infection in dogs, based on information collected from studies published between 2015 and 2024 across 14 European countries. Available data was compiled and analyzed following PRISMA guidelines, revealing pronounced geographical variability. Prevalence was highest in southern European countries such as Portugal, Italy, and Greece, while substantially lower rates were reported in Central and Northern Europe. Despite differences in diagnostic approaches and surveillance intensity across countries, overall patterns point to endemic transmission in southern regions and a growing epidemiological risk in areas traditionally considered non-endemic. These findings underscore the need for harmonized diagnostic protocols and a strengthened surveillance network, consistent with the One Health framework, to enable effective responses to climate change and the shifting landscape of vector-borne diseases in Europe.

## 1. Introduction

Leishmaniasis is a disease caused by protozoa of the family Trypanosomatidae, genus *Leishmania*, and is transmitted by vectors. The disease is increasingly reported in both animal and human populations worldwide [1,2]. Its particular significance in veterinary medicine lies in the wide range of susceptible animal hosts, as well as in the abundance and diversity of vectors present in the environment. In the host organism, the parasites exhibit an intracellular localization, primarily within macrophages of the phagocytic system [3,4]. To date, approximately 30 *Leishmania* species have been described, of which 20 are pathogenic to mammals [5] and 18 of these pathogenic species also possess zoonotic potential [6].

The vectors responsible for the transmission and spread of this disease are dipterans belonging to the *Phlebotomus* and *Sergentomyia* (Old World) genera, as well as *Lutzomyia*, *Brumptomyia*, and *Warileya* (New World). These insects are collectively referred to as sand flies, and transmission occurs through the bites of infected hematophagous females [7,8,9,10].

Wild animals such as rodents, hyraxes, and wild carnivores were originally considered the primary reservoirs of the pathogen. However, following the domestication of animals and continuing to the present day, the dog has emerged as the most important host and reservoir, playing a central role in the epidemiology of the disease [11,12].

Canine leishmaniasis is currently reported in nearly 90 countries across five continents, with particularly high occurrence in China, Brazil, and the Mediterranean Basin. Its geographical distribution is closely tied to the presence of competent vectors, and in recent years has expanded due to climate change, which has created favorable conditions for the completion of the vectors’ life cycle [13,14].

Risk factors such as increased migration, widespread deforestation, urbanization, malnutrition, immunosuppression, and unsuccessful treatments have contributed to the global rise in leishmaniasis in both humans and animals [6].

In Europe, the population of companion animals has grown and diversified considerably in recent years. Dogs and cats now play a more significant social role due to the close human–animal bond. As a result, an increasing number of companion animals receive a high level of care, live in close proximity to humans, and often travel alongside them. These animals are central to the evolving One Health framework, highlighting their importance in the dynamics of both veterinary and human leishmaniasis. Although several European countries have implemented surveillance programs, standardized reporting and cross-border data integration are still insufficient to accurately estimate the true burden of the disease [15].

Depending on the *Leishmania* species involved, the host’s immune profile, and the clinical presentation, leishmaniasis has three main clinical manifestations: cutaneous, mucocutaneous, and visceral. The visceral form is the most common and the most severe, frequently leading to fatal outcomes. In dogs, clinical signs include cutaneous lesions, weight loss, anorexia, lymphadenopathy, ocular lesions, epistaxis, locomotor dysfunction, and muscle atrophy [16,17,18].

Diagnosis of leishmaniasis is challenging when based solely on anamnesis and clinical signs, given the wide variability of presentations. The most reliable approaches for establishing a definitive diagnosis include serological tests to detect humoral and cellular immune responses, as well as microscopic identification of the parasite [4,19,20].

Considering the growing number of epidemiological studies on canine leishmaniasis in Europe over the past decade, there is a clear need for an updated synthesis that highlights the geographical distribution of the disease, reported prevalence rates, and diagnostic methods employed. The aim of this review is to comparatively analyze data published between 2015 and 2024, with particular focus on prevalence related regional variability, applied diagnostic techniques, and the identified *Leishmania* species. By consolidating these findings, the review seeks to provide an integrated overview of the current status of canine leishmaniasis in Europe and to enhance the understanding of factors shaping its distribution in order to support more effective monitoring and control strategies at both regional and continental levels.

## 2. Materials and Methods

This systematic review was conducted in accordance with the PRISMA (Preferred Reporting Items for Systematic Reviews and Meta-Analyses) guidelines and aimed to synthesize data regarding geographical distribution and prevalence of canine leishmaniasis in Europe between 2015 and 2024 [21]. This systematic review adheres to the PRISMA 2020 guidelines, and all items from the PRISMA checklist have been completed and can be found in the Supplementary Materials.

Literature research was carried out between June and July 2025 using Google Scholar, Web of Science, and PubMed. The search strategy combined the following terms with Boolean operators (AND/OR): “leishmaniasis,” “canine,” “dog,” and “Europe.” The time frame covered publications published from 1 January 2015 to 31 December 2024. At the initial stage, no restrictions were applied regarding the employed diagnostic methods or the characteristics of the studied canine populations.

Inclusion criteria were:

- original studies published between 2015 and 2024;
- studies conducted in Europe (specifically, in European countries or territories);
- explicit reporting of *Leishmania* spp. prevalence in dogs;
- specification of the employed diagnostic method and the type of sample;
- identification of *Leishmania* species involved, where possible.

Exclusion criteria consisted of review type studies, experimental studies without an epidemiological component, case reports, studies not available in full text, studies conducted outside of Europe, studies reporting only aggregated multi-regional results without stratification for Europe, and studies from transcontinental countries where the exact location could not be clearly determined. The geographical delimitation of Europe followed the United Nations (M49) classification. Countries located entirely within continental Europe were included, and Cyprus was considered part of Europe. Studies from Turkey were excluded, as all available data originated from the Asian region. No language restrictions were applied. From each article included in the review, key information was extracted and compiled into a summary table: the country where the study was conducted, the type of biological samples analyzed, the diagnostic method employed, the *Leishmania* species identified, the total number of dogs tested, the number of positive samples, the reported prevalence, and the bibliographic reference.

The risk of bias and methodological quality of the included studies were evaluated through a standardized critical appraisal, following PRISMA recommendations. Particular attention was given to the clarity of methodological descriptions, sample size, and the accuracy of the reported results.

Statistical analyses and graphical outputs were generated using Excel and Map Chart to produce comparative figures and thematic maps. To allow cross-country comparisons, a weighted national mean prevalence of canine leishmaniasis was calculated, incorporating both the number of positive cases and the sample size reported in eligible studies. When the same canine population was tested by multiple diagnostic methods within a single study, only one prevalence value was retained—either corresponding to the main method reported or to the largest sample size—in order to avoid duplication.

For each prevalence estimate, 95% confidence intervals (95% CI) were calculated using the formula p ± 1.96 × √[p(1 − p)/n], where p denotes the proportion of positive dogs and n the sample size. Results are presented as percentages with corresponding confidence limits and visualized with error bars.

## 3. Results

### 3.1. Study Selection Process

**Figure 1:**
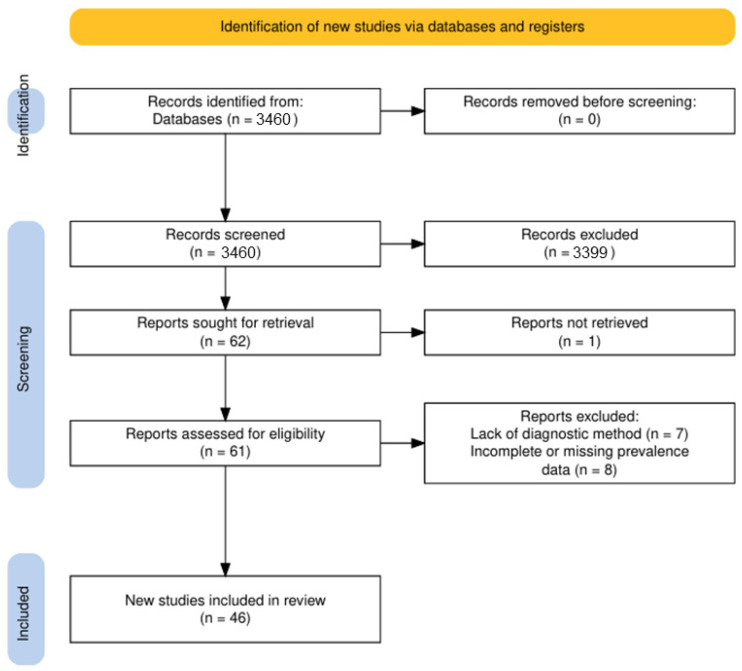
PRISMA 2020 flow diagram illustrating the selection of studies on canine leishmaniasis in Europe (2015–2024) [21].

The article selection process is illustrated in detail in the PRISMA flow diagram (Figure 1) [21]. In total, 3460 records were identified through database searches: 3160 from Google Scholar, 210 from PubMed, and 90 from Web of Science.

After removal of duplicates and screening of titles and abstracts, 61 articles were assessed in full text. Ultimately, 46 studies met the inclusion criteria and were retained for detailed analysis. A summary of the extracted data from these 46 studies is presented in Table 1.

### 3.2. Geographical Distribution and Prevalence

The studies included in the review covered 14 European countries. Reported prevalence of *Leishmania* spp. infection ranged from 0% to 60.4%, reflecting marked epidemiological heterogeneity across the continent. The highest prevalence rates were observed in southern European countries: Portugal (60.4%), Italy (54.13%), Greece (over 20%), and North Macedonia (25%). By contrast, much lower prevalence values were reported in Central and Eastern Europe, including Poland (0.2%), Slovenia (1.9%), and Croatia (1.38%).

### 3.3. Weighted Means Prevalence

**Figure 2:**
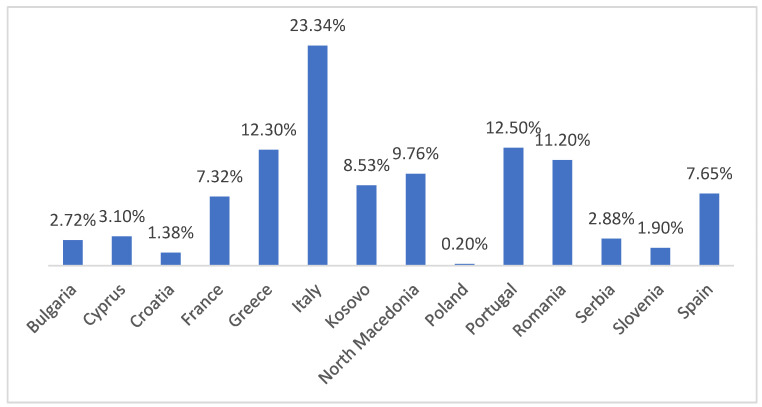
Prevalence of weighted means.

The weighted mean prevalence was calculated for each country using sample-size weighting for all included studies. This approach provides a more accurate and representative estimate of prevalence by reducing the disproportionate influence of small-sample studies or those reporting extreme values. Weighted prevalence ranged from 0.2% in Poland to 23.34% in Italy, reflecting the true epidemiological impact of canine leishmaniasis across different European countries. The exact weighted prevalence values used to generate the thematic map are presented in detail in Figure 2.

**Figure 3:**
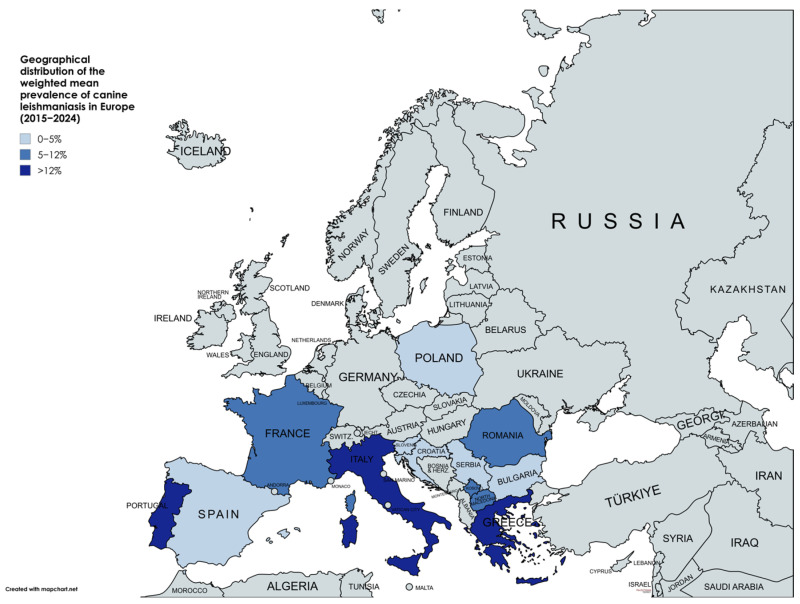
Geographical distribution of the weighted mean prevalence of canine leishmaniasis in Europe (2015–2024) [68].

Figure 3 shows the thematic map of the distribution of the weighted mean prevalence of *Leishmania* spp. infection in dogs across the European countries included in this review.

### 3.4. Confidence Intervals

**Figure 4:**
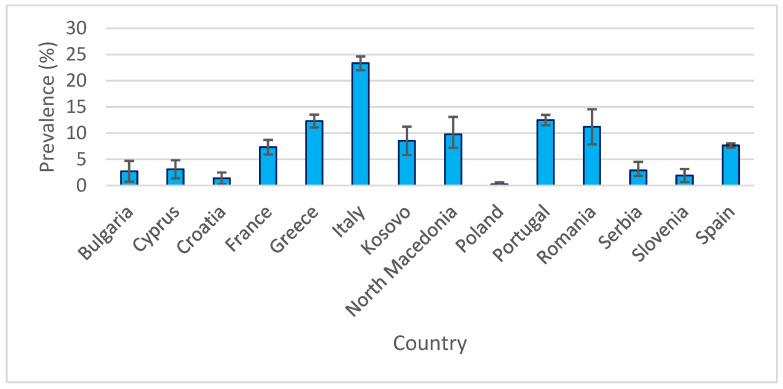
Weighted mean prevalence (95% CI) of canine leishmaniasis in 14 European countries (2015–2024).

National prevalence estimates of canine leishmaniasis, accompanied by 95% confidence intervals (95% CI), revealed substantial variation between countries. The lowest values were recorded in Poland (0.20%; 95% CI: 0.00–0.59%) and Croatia (1.38%; 95% CI: 0.28–2.48%), whereas the highest were reported in Italy (23.34%; 95% CI: 22.02–24.66%) and Greece (12.3%; 95% CI: 11.07–13.53%). Countries with large sample sizes, such as Portugal (n = 4294), yielded narrower confidence intervals, indicating greater precision in prevalence estimates. In contrast, countries with smaller samples, such as Kosovo (n = 410; prevalence 8.53%; 95% CI: 5.83–11.23%), showed wider intervals, reflecting greater uncertainty. A graphical representation of these results (Figure 4) precisely illustrates the variability across countries, as indicated by error bars.

### 3.5. Relationship Between the Size of the Cohort and Prevalence

Graphical analysis of the relationship between sample size and the reported prevalence of *Leishmania* spp. infection in dogs showed a weak negative trend. Smaller-sample studies tended to report higher prevalence rates on average compared with larger-scale studies. This phenomenon may be partly explained by potential selection bias, in which study populations more frequently include dogs at higher risk of infection.

**Figure 5:**
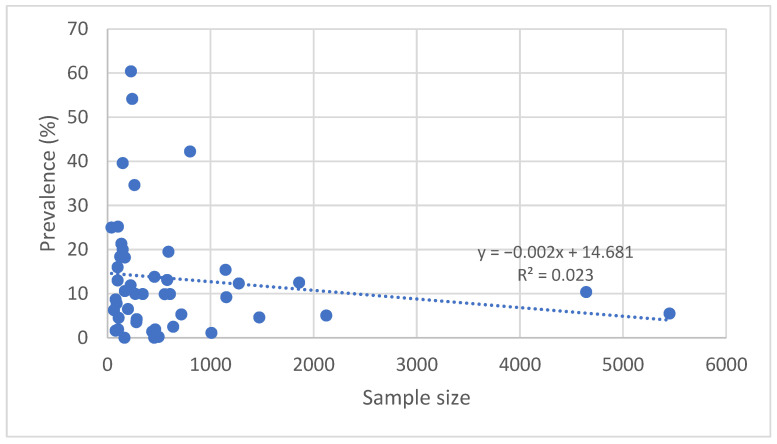
Relationship between sample size and reported prevalence of canine leishmaniasis in Europe (2015–2024).

The coefficient of determination (R^2^) for this relationship was low, indicating that sample size alone does not fully account for the variation in reported prevalence. Nevertheless, this observation highlights the importance of using representative samples and conducting multicenter studies to obtain more accurate estimates. Figure 5 illustrates this relationship, with each point representing an individual study and the dashed line indicating the linear regression.

### 3.6. The Influence of Diagnostic Methods on Prevalence Rate Calculations

**Figure 6:**
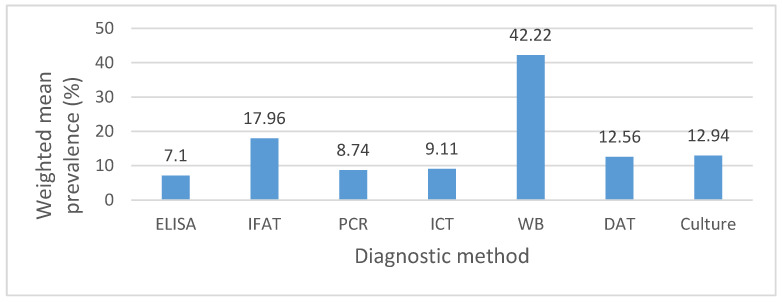
Weighted mean prevalence of canine leishmaniasis in Europe (2015–2024) according to the diagnostic method used.

Comparative analysis of the data revealed significant differences between the diagnostic methods used across the included studies. Serological tests such as IFAT and ELISA reported prevalence rates ranging from 0% to 54.13%, while molecular methods (PCR) showed a wider variation from 0% to 60.4%. Western Blot, applied in a single study, yielded a high prevalence of 42.22%, whereas rapid tests (ICT) reported moderate values ranging from 0% to 25%. Culture, although less frequently applied, demonstrated a weighted mean prevalence of 12.94%, comparable to DAT (12.56%), suggesting that parasitological confirmation methods tend to yield intermediate prevalence estimates. Figure 6 illustrates the comparison of weighted mean prevalence across the main diagnostic approaches, highlighting that molecular methods tend to report higher values, while rapid tests produce lower estimates. These findings emphasize the influence of the chosen diagnostic method on the prevalence outcomes reported.

## 4. Discussion

### 4.1. Geographical Variation in Prevalence

Findings indicate that the weighted mean prevalence of canine leishmaniasis in Europe follows a distinct geographical pattern, with higher values in southern Mediterranean countries such as Italy, Portugal, and Greece. In Italy, canine leishmaniasis remains an endemic disease, though with a heterogeneous geographical distribution that now includes not only traditionally endemic southern regions but also areas previously considered non-endemic. For example, in the province of Bolzano, located in the eastern Alps and historically regarded as non-endemic, autochthonous cases (7.7%) confirmed by molecular methods have been reported, suggesting low-level local transmission [33]. In southern Italy, particularly in Apulia, both *L. infantum* and *L. tarentolae* infections have been identified, highlighting the complex ecology of the parasite in this region [36]. High prevalence rates have also been recorded in insular regions, such as the Aeolian Islands (41.8%—[38]) and Pantelleria Island (27%—[37]). In Sardinia, the reported seroprevalence (15.4%) was comparable to that of continental regions, confirming the endemic nature of the disease [35].

Findings from earlier literature support these observations. Ferroglio et al. investigated dogs and sand flies from northern Italian regions between January 1999 and March 2001, reporting seropositive dogs originating from areas where the disease had not previously been documented, along with the presence of more than 5000 sand flies from four different species. These results suggested that northward expansion of the disease was already underway at the beginning of the 21st century [69].

In Greece, an epidemiological survey reported an average seroprevalence of 22.09% among 5772 randomly selected dogs from 43 prefectures [70]. Similarly, around the same time as the Italian studies, Ivovic et al. identified 10 sand fly species in both continental and insular parts of Greece [71]. Taken together, these data reinforce the view that canine leishmaniasis is endemic in these countries while simultaneously undergoing significant geographic expansion.

Within the Eastern Mediterranean context, Cyprus also presents evidence of low-level endemic transmission, with marked local variation. Historical data indicate long-standing circulation of *L. infantum*: in 1996, the southern part of the island recorded a seroprevalence of 1.7% in a general sample (n = 601) and 10% in selected groups from outbreak areas (n = 301), with local maxima of 26.2% in Agios Georgios and 12.2% in Limnatis [72]. In the northern region, studies conducted in two investigation periods reported 3.61% positivity in 2004 and 3 of 5 dogs testing positive in clinical cases in 2012. At the same time, nine sand fly species were identified, although no parasites were detected in vector samples. The authors concluded that the risk of canine leishmaniasis was increasing in Northern Cyprus [73].

Subsequent studies, however, reported low prevalence levels in the north, at 1.9% and 3.55% [24,25]. Overall, reported cases remain relatively few, likely due to the combined effects of insularity (with limited canine mobility), urbanization of sampled areas, and adoption of prophylactic measures, against a background of uneven vector pressure that generates only localized foci in the north.

France, Portugal, and Spain—neighboring countries from southwestern Europe and all part of the Mediterranean Basin—show variable levels of *L. infantum* positivity in canine populations, reflecting local epidemiological characteristics. In France, studies conducted in the southeast and in the Marseille area reported seroprevalence rates ranging from 5.3% to 9.9%, with concurrent detection of parasitic DNA in canine blood, indicating both past exposure and active infections [27,28].

In Portugal, prevalence rates were generally higher, ranging from 9.9% to 18.2% depending on the region and category of studied dogs, confirming the well-established endemicity of the disease in the country [48,49,51]. Moreover, in southern Portugal, the presence of competent vectors has been confirmed through morphological and molecular identification of *Phlebotomus perniciosus* and *Sergentomyia minuta* infected with *L. infantum* [51], further supporting the stable endemic status of the disease in this region.

Marked heterogeneity is particularly evident in Spain, where regional studies have reported high prevalence of canine leishmaniasis—for example, 19.5% in Girona province [67]—while large-scale investigations conducted on dogs with owners have yielded much lower values, around 5% [63]. This intranational variability reflects substantial regional differences, likely driven by climatic factors, vector density and distribution, the type of studied canine populations, and the extent of prophylactic measures applied. At a broader scale, the heterogeneity observed among Spain, Portugal, and France can be attributed to similar factors, compounded by local epidemiological specificities, underscoring the need for surveillance and control programs tailored to each geographical context.

In Romania (11.2%) and North Macedonia (9.76%), the weighted mean prevalence estimates obtained during the study period were comparable, indicating significant circulation of the parasite in both countries. In North Macedonia, surveys conducted in the Tetovo region [44] and in other areas [45,46] have seropositivity rates ranging from 1.6% to 25%, confirming active parasite presence in local canine populations. In Romania, investigations carried out in different regions—including Râmnicu Vâlcea [55]), Argeș [56], Galați and Călărași [57]—also demonstrated the presence of *L. infantum* DNA or specific antibodies, with prevalence values ranging from 3.7% to over 20%.

It is noteworthy that the presence of vectors (*Phlebotomus* spp.) in Romania has been previously documented [74,75], confirming the existence of conditions conducive to local transmission. These findings support the prerogative that in both Romania and North Macedonia, canine leishmaniasis is or has great potential to become endemic, with a risk of further expansion in the absence of surveillance and control measures tailored to local contexts.

In Bulgaria (2.72%) and Serbia (2.88%), available epidemiological data are limited and suggest a relatively low circulation of the parasite. In Bulgaria, earlier studies did not identify seropositive cases [23], while more recent investigations have reported the parasite only in isolated cases [22]. In Serbia, a survey of 455 dogs from various regions did not detect any positives [46], whereas another study conducted in the north of the country reported a prevalence of 10.59% [58]. This discrepancy likely reflects regional differences in parasite distribution and highlights the need to expand monitoring in these two neighboring countries, both located in proximity to endemic areas of southern Europe.

Weighted prevalence estimates place countries such as Poland (0.2%), Croatia (1.38%), and Slovenia (1.9%) at the lower end of the canine leishmaniasis spectrum according to literature published within the study period. Although data on canine leishmaniasis are limited in Poland, a study conducted between 2017 and 2023 reported a seropositivity rate of 1.17% in European bison (*Bison bonasus*). This finding suggests parasite circulation in regions previously regarded as non-endemic, as well as in wildlife populations. The detection of seropositivity in such an emblematic wild species underscores the need to expand surveillance beyond domestic dogs to include other susceptible hosts, in order to better understand parasite dynamics and the risk of spread into new regions [76]. Although Croatia and Slovenia both have a coastline along the Adriatic Sea, part of the Mediterranean Basin, the overall climatic influence across their territories is limited, which may partly explain the low prevalence rates reported in these neighboring countries. Between 2002 and 2004, 2917 sand fly specimens from two genera (*Phlebotomus* and *Sergentomyia*) were collected in southern Croatia, already indicating the potential for disease emergence. Later, between 2007 and 2009, testing of 2035 immunocompetent individuals without clinical signs revealed a seropositivity rate of 11.4%, pointing to significant exposure to *Leishmania* spp. in the human population. These findings suggest the existence of subclinical transmission and active parasite circulation, even in the absence of overt clinical disease [77].

In Slovenia, the first study conducted on autochthonous dogs reported a seroprevalence of 1.9% through ELISA, while all samples tested negative through PCR and IFAT. Interestingly, more than half of the seropositive dogs had no travel history to endemic areas, suggesting the possibility of local transmission and, consequently, the risk of emerging endemic foci [59].

Within the Balkan region, Kosovo presents a profile of modest national prevalence but with local hotspots and recent entomological evidence supporting autochthonous circulation of the vectors and parasites. Following the resumption of entomological surveys in 2014, nine sand fly species were identified, including newly recorded species for the country, and the detection of *L. tropica* in *Ph. neglectus* confirmed the high vector potential [78]. A subsequent nationwide survey in 2022, covering all seven districts, documented—for the first time—the presence of *L. infantum* DNA in *Ph. neglectus* and *Ph. perfiliewi* [79]. Canine data are consistent with this picture of localized yet spatially heterogeneous transmission: in the southwest, a 2020 study reported 18.4% seropositivity with few clinical cases [43], whereas a nationwide survey conducted between summer 2021 and spring 2022 estimated 4.21% positivity by ELISA and 3.51% by IFAT among asymptomatic dogs [42]. Taken together, the diversity and presence of sand fly vectors, combined with detection of *L. infantum* in field-caught specimens, explain the occurrence of positive canine cases and underscore the need for integrated dog–vector surveillance and targeted interventions in areas of risk.

Southern Europe offers favorable conditions for maintaining the parasite’s life cycle, including warmer temperatures, lower altitudes, and rural or peri-urban environments with high dog density [80]. Medlock et al. reported an increasing risk of sand fly establishment in areas outside their traditional range, including the Atlantic coast and inland regions of Germany, Switzerland, and Austria. In addition to areas already considered suitable for vectors, the authors identified further regions with colonization potential, suggesting that sand flies may already be present over wider areas than previously reported [81].

### 4.2. Impact of Diagnostic Methods

Estimates of canine leishmaniasis prevalence are strongly influenced by diagnostic choices and their implementation, including the type of test, positivity threshold, confirmation algorithm, and the structure of the studied population. In our dataset, the apparent differences between countries or regions are more likely explained by these methodological variations than by true differences in transmission.

Serological tests capture evidence of immunological exposure, whereas direct detection methods (qPCR/isolation) provide a better indication of active infection or detectable parasitemia [82]. Among the analyzed studies, the relationship between the two varied predictably depending on context (endemicity level, dog status, sample type), producing patterns of concordance and discordance that are useful for comparative interpretation.

A large survey conducted in Marseille (France), between May and October 2023 (n = 718) found a seroprevalence of 5.3% and a blood qPCR positivity of 3.2%. Among seropositive dogs, 59% were also qPCR-positive (22/37), although discordant results were observed (one qPCR-positive/seronegative dog; 14 seropositive/qPCR-negative dogs). This profile indicates ongoing transmission, with detectable parasitemia in only a subset of exposed dogs, as typically expected in moderately endemic settings [27]. By comparison, in a Romanian shelter study of asymptomatic dogs, qPCR detected *L. infantum* DNA in 20.1% (30/149), while all animals tested seronegative, suggesting that in early stages of infection, or in clinically healthy hosts, reliance on serology alone may underestimate prevalence [56].

In Slovenia, 1.9% of dogs tested seropositive on ELISA, yet none were positive for the blood or conjunctival swab qPCR. This pattern (“serology > qPCR” at low prevalence) is consistent with past exposure, parasite loads below the detection threshold, or tissue distribution not captured by the sampled matrices. Such findings justify cautious interpretation of low seroprevalence values [59].

The Mediterranean Island landscape clearly illustrates the effect of diagnostic methodology. In the Aeolian Islands, serology in dogs indicated widespread exposure (≈35–42%), whereas direct detection in blood captured only a fraction of infections (≈9%), with higher molecular yields obtained from conjunctival secretions (≈12%). These differences reflect both the cumulative nature of the humoral response and the critical importance of sample choice for qPCR [38]. In the Pelagie Islands, overall canine positivity was similar (≈39.6%), but *L. infantum*-specific qPCR from blood yielded much lower rates; here, serology reflects exposure pressure, while molecular detection confirms active infection. This supports a two-step diagnostic algorithm and the use of appropriate sample types for direct detection [40].

A very similar profile was recently reported in Crete: among 77 serologically tested dogs, most exhibited high titers (58.4% at 1/1600), indicating intense exposure, while qPCR positivity was substantially higher from conjunctival secretions (91.9%) compared with blood (79.1%) [83]. These findings confirm that the choice of sample type decisively influences the sensitivity of molecular detection and that, in high-transmission areas, combining serology with qPCR provides a much more accurate picture of true prevalence.

Even within serological testing, notable differences arise: in the nationwide survey conducted in Kosovo (summer 2021–spring 2022), ELISA estimated prevalence at 4.21% (95% CI: 2.42–7.21), while IFAT yielded 3.51% (95% CI: 1.92–6.34), against a background of marked spatial heterogeneity (8.0%/6.0% in Prishtina vs. 0% in Mitrovica). Such test-related variability highlights the importance of reporting both results in parallel or applying a standardized diagnostic algorithm to ensure robust comparisons across studies [79].

### 4.3. Relationship Between Sample Size and Prevalence

A clear trend in our dataset is the weak negative correlation between sample size and estimated prevalence: studies with large numbers of dogs and broad geographical coverage tend to yield moderate prevalence values with narrower confidence intervals, whereas smaller studies, often focused on hotspots or high-risk populations, more frequently report elevated prevalence rates. This phenomenon is well recognized in epidemiological research, as the precision of estimates increases proportionally with the square root of the sample size [84,85].

Recent literature provides examples supporting this observation. In Kosovo, a regional study of 125 dogs reported a prevalence of 18.4%, while a nationwide survey of 285 asymptomatic dogs across seven districts produced lower estimates of 4.21% by ELISA and 3.51% by IFAT, with notable spatial contrasts between Pristina (8%/6%) and Mi-trovica (0%) [42,43]. In France, a large-scale population survey in Marseille (718 dogs) reported moderate, well-defined prevalence rates (5.3% serology; 3.2% qPCR) with narrow confidence intervals, illustrating the advantages of robust study design [27].

At the opposite extreme, in Romania, a study of 149 shelter dogs found 20.1% qPCR positivity alongside 0% seropositivity, a pattern consistent with either early stages of infection or selection bias in the studied population [56]. In Slovenia, a larger survey of 465 dogs reported a low and stable prevalence (1.9% serology; 0% qPCR), consistent with low transmission pressure and a representative sample [59].

Taken together, these examples demonstrate that small-sample studies often reflect local realities or focal situations, whereas large, geographically distributed surveys provide more robust and comparable benchmarks across regions. This observation has important implications for the critical interpretation of literature and for the design of monitoring strategies.

### 4.4. Future Directions

To obtain robust and comparable prevalence estimates, future studies should adopt standardized diagnostic and reporting protocols. These should explicitly specify the diagnostic test employed (e.g., ELISA, IFAT, qPCR), clearly define positivity thresholds, apply a two-step algorithm (serological screening followed by molecular confirmation), indicate the type of sample that was analyzed (e.g., blood vs. conjunctival swabs), and consistently report confidence intervals stratified by clinical status (asymptomatic vs. symptomatic), dog type (shelter vs. owned), and environment of origin (urban vs. rural).

Large-scale, multicenter studies with balanced geographical coverage are needed to capture the full European gradient, including northern regions where data remain scarce but where shifts in disease distribution may occur. Sampling should also be seasonally synchronized and paired with parallel entomological surveillance, enabling correlations between canine prevalence, vector abundance and vector dynamics.

At ecosystem level, international collaboration is essential. Common protocols and standard operating procedures (SOPs), inter-laboratory testing and external quality assessment (EQA), open-access data registries, and shared infrastructures for meta-analyses and geographic information systems (GIS) would ensure reproducible, interoperable results that can be directly translated into effective control policies.

## 5. Conclusions

Canine leishmaniasis remains a major concern for both public and veterinary health in Southern Europe, where high prevalence rates and endemic transmission persist. In contrast, Northern regions continue to report very low prevalence rates; however, the potential impact of climate change cannot be overlooked, as it may increase vulnerability in currently non-endemic areas in the near future.

The findings of this review highlight the need for standardized diagnostic approaches, the implementation of large-scale multicenter studies, and strengthened international collaboration to support effective surveillance and control strategies.

## Figures and Tables

**Table 1:** Epidemiological evidence of canine leishmaniosis across European countries (2015–2024).

No.	Country	Sample	Diag. Method	Species	No. of Samples	Positive Samples	Prevalence	Ref.
1.	Bulgaria	Serum	ELISA	*L. infantum*	90	7	7.77%	[22]
		Serum	Elisa	No. sp.	167	0	0%	[23]
2.	Cyprus	Serum	IFAT	*L. infantum*	105	2	1.9%	[24]
		Serum	IFAT	*L. infantum*	281	10	3.55%	[25]
3.	Croatia	Serum	IFAT	*L. infantum*	435	6	1.38%	[26]
4.	France	Serum	ELISA	*L. infantum*	718	37	5.3%	[27]
		Blood	PCR			23	3.2%	
		Serum	ELISA	*L. infantum*	607	60	9.9%	[28]
5.	Greece	Serum	ELISA	*Leishmania* spp.	1154	106	9.2%	[29]
		Blood	Culture	*Leishmania* spp.	1275	165	12.94%	[30]
		Serum	ELISA	*L. infantum*	103	26	25.2%	[31]
		Blood	PCR	*L. infantum*	200	13	6.5%	[32]
6.	Italy	Serum	IFAT	*L. infantum*	457	63	13.8%	[33]
		Serum	IFAT	*L. infantum*	242	130	54.13%	[34]
		Serum	IFAT	*L. infantum*	1147	176	15.4%	[35]
		Serum	IFAT	*L. infantum*, *L. tarentolae*	100	16	16%	[36]
		Lymph nodes	PCR	*L. infantum*	136	29	21.32%	[37]
		Serum	IFAT	*L. infantum*	263	91	34.6%	[38]
		Conj. swab	PCR			32	12.2%	
		Blood				24	9.1%	
		Serum	IFAT	*L. infantum*	639	16	2.5%	[39]
		Serum	IFAT	*Leishmania* spp.	149	59	39.6%	[40]
		Blood	PCR			5	2.98%	
		Serum	WB	*L. infantum*	803	339	42.22%	[41]
7.	Kosovo	Serum	ELISA	*L. infantum*	285	12	4.21%	[42]
			IFAT			10	3.51%	
		Serum	ELISA	*L. infantum*	125	23	18.4%	[43]
8.	North Macedonia	Blood	ICT	*L. infantum*	272	27	9.92%	[44]
			ICT	*L. infantum*	40	10	25%	[45]
			PCR	*L. infantum*	80	2	1.6%	[46]
9.	Poland	Blood	PCR	*L. infantum*	497	1	0.2%	[47]
10.	Portugal	Serum	DAT	*Leishmania* spp.	343	34	9.9%	[48]
		Serum	DAT	*L. infantum*	1860	233	12.5%	[49]
		Blood	PCR	*L. infantum*	1010	11	1.1%	[50]
		Blood	DAT	*L. infantum*	170	31	18.2%	[51]
		Serum	ELISA	*L. infantum*	100	13	13%	[52]
		Serum	ELISA	*Leishmania* spp.	581	76	13.1%	[53]
		Blood	PCR	*L. infantum*	230	139	60.4%	[54]
11.	Romania	Serum	ELISA	*L. infantum*	80	3	3.7%	[55]
		Conj. swab	PCR	*Leishmania* spp.		7	8.7%	
		Blood				1	1.2%	
		Blood, conj. swab	PCR	*L. infantum*	149	30	20.1%	[56]
		Serum	ICT	-		0	0%	
		Serum	ELISA	*Leishmania* spp.	110	5	4.54%	[57]
12.	Serbia	Blood	PCR	-	455	0	0%	[46]
		Serum	ELISA	*L. infantum*	170	18	10.59%	[58]
13.	Slovenia	Serum	ELISA	*Leishmania* spp.	465	9	1.9%	[59]
		Conj. swab, blood	PCR			0	0%	
14.	Spain	Serum	ELISA	*Leishmania* spp.	226	27	11.9%	[60]
		Blood	PCR	*L. infantum*	65	4	6.25%	[61]
		Serum	ICT	*L. infantum*	4643	481	10.36%	[62]
		Serum	IFAT	*L. infantum*	2123	107	5.05%	[63]
		Blood	ICT	*L. infantum*	1474	68	4.61%	[64]
		Serum	ELISA	*L. infantum*	556	55	9.88%	[65]
		Serum	ELISA	*L. infantum*	5451	300	5.5%	[66]
		Serum	ELISA	*L. infantum*	593	116	19.5%	[67]

Legend: IFAT—Indirect Fluorescent Antibody Test; ELISA—Enzyme-Linked Immunosorbent Assay; PCR—Polymerase Chain Reaction; DAT—Direct Agglutination Test; ICT—Immunochromatographic Test; WB—Western Blot.

## Data Availability

Data are contained within the article.

## Supplementary Materials

The following supporting information can be downloaded at: https://www.mdpi.com/article/10.3390/pathogens14111082/s1, Table S1: PRISMA 2020 checklist. Reference [86] is cited in the supplementary materials.
